# Evaluating the contribution of antimicrobial use in farmed animals to global antimicrobial resistance in humans

**DOI:** 10.1016/j.onehlt.2023.100647

**Published:** 2023-10-31

**Authors:** Zahra Ardakani, Massimo Canali, Maurizio Aragrande, Laura Tomassone, Margarida Simoes, Agnese Balzani, Caetano Luiz Beber

**Affiliations:** a Department of Agricultural and Food Science, University of Bologna, Italy; b Department of Veterinary Sciences, University of Turin, Italy; c Comprehensive Health Research Centre (CHRC), University of Evora, Portugal; d Animals in Farming Programme, World Animal Protection, United Kingdom

**Keywords:** Antimicrobial resistance (AMR) in humans, Antimicrobial use (AMU) in farmed animals, Antibiotics, Spatial analysis, One health

## Abstract

Antimicrobial resistance (AMR) is currently regarded by the World Health Organization (WHO) as one of the most significant risks to global public health. The most critical causes of AMR infections in humans are the misuse and overuse of antimicrobials in humans and farmed animals. The rising global demand for food of animal origin encourages the increase of animal production worldwide, especially in developing countries. Simultaneously, current farming practices often extensively use antimicrobials on animals, influencing bacterial AMR incidence. This study aims to evaluate the correlation between antimicrobial use (AMU) in farmed animals and the detection of AMR infections in humans, the effects of enforcing laws in animal farming in a country on AMR situation in the neighbors, and the potential of AMR to spread from one country to another. Using data from 30 largest animal-producing countries in different regions of the world, between 2010 and 2020, and a Spatial Durbin Model (SDM), we found that AMU in farmed animals increases AMR in humans and there is a spatial dependence between countries regarding AMR spreading. Such findings indicate that a globally coordinated strategy regulating AMU on farmed animals may reduce AMR emergence and worldwide spreading.

## Highlights

• Antimicrobial use (AMU) in farmed animals is positively correlated with antimicrobial-resistance (AMR) in humans.
• There is a spatial dependence between countries regarding the spread of AMR infections in humans.
• A globally coordinated strategy regulating AMU in farmed animals contributes to AMR reduction at a global scale.

## 1 Introduction

An increasing number of infections will become more challenging to treat and, in certain cases, even impossible if antibiotics lose their effectiveness [1]. Antimicrobial resistance (AMR) is currently identified by the World Health Organization (WHO) as one of the most severe global threats to public health [2] and the economy [3]. AMR bacteria are found in humans, animals, food, and the environment (including air, water, and soil) and can be transmitted between them [4]. AMR is a complex problem with multiple drivers [5], and inappropriate antimicrobial use (AMU) in both humans and animals may be the primary cause of its rapid increase and spread [6,7] globally.

The growing demand for food of animal origin [8] has contributed to increasing animal farming globally [[9], [10], [11]], where antimicrobials are critical in treating animals' diseases [4]. The farmed animals are treated with antibiotics not only for therapeutic purposes but also for prophylaxis, metaphylaxis, and growth promotion to reduce animal health risks and maximize productivity [12]. Such practices contribute to developing resistant bacteria affecting animals and humans [7,13], and making it feasible for resistant microorganisms to spread quickly and silently in a globally interconnected world [5,13,14].

Modern animal farming faces the dual challenge of increasing production and combating antimicrobial resistance (AMR) due to the ongoing global demand for food of animal origin. [15]. While creating new markets for farmed animals, globalization and international trade have facilitated the spread of foodborne infectious diseases [16] that can also carry resistant bacteria across international borders [17]. Moreover, global displacements of persons contribute to the spread of resistant bacteria: it was estimated that, over the last decade, 30% of international travelers were in contact with resistant bacteria [17,18].

The literature agrees that the inappropriate AMU in farmed animals contributes to AMR in humans. Still, the details and size of this connection and how it is influenced by other factors, such as current AMR levels and social, economic, and epidemiological factors, need to be unraveled [19]. Most studies primarily analyze data from high-income countries with similar socioeconomic conditions. Nonetheless, the lack of data from low- and middle-income countries hinders evaluating the impact of critical parameter changes [19]. For example, in a recently published paper, the authors calculated the effect of AMU in farmed animals on the prevalence of AMR in humans (a 1% increase in AMU in farmed animals increases AMR in humans between 0.03% and 0.40%) but only for the European Union (EU) countries. They also ignored other factors (e.g., environmental factors) that affect AMR in humans and animals [20].

Therefore, correctly understanding the connection between AMU in farmed animals and AMR in humans is crucial for acting and safeguarding antibiotic efficacy [19] and reducing the related global public health costs [21,22]. In this paper, we address the critical gaps highlighted by Emes et al., [19] and Rahman and Hollis [20] regarding the factors influencing AMR in humans. We use the WHO framework as a comprehensive tool to capture the complexity of AMR drivers, avoiding the bias caused by omitting relevant variables. We also include data from high-income and low- and middle-income countries in our analysis to account for the global nature of AMR. Moreover, we apply spatial methods to investigate the cross-border transmission of AMR among countries, which can also reveal the effect of current AMR levels on future trends.

We selected nine animal species that are the primary sources of animal food for humans worldwide: cattle, chickens, pigs, carp, catfish, salmons, shrimps, tilapias, and trout [23,24]. From the disease perspective, we chose two bacteria that cause most of the foodborne illnesses and AMR infections in humans: *Escherichia coli* (*E. coli)* and *Staphylococcus aureus (S. aureus)* [[25], [26], [27], [28]]. Then, by using data from thirty large producer countries from different regions of the world and covering the period from 2010 to 2020, this paper aims to address three main questions:

1. How does AMU on farmed animals in a country affect AMR in humans in that country?
2. How do policies regarding AMU on farmed animals in one country influence AMR in humans in neighboring countries?
3. How does AMR in humans cross over national borders?

## 2 Material and methods

This section provides an overview of our method, followed by the data we collected and analyzed.

### 2.1 Spatial analysis

Non-spatial regression models assume that observations are independent [29], but this assumption needs increased accuracy, and there may be dependencies between observations at several locations or regions [30]. Spatial models allow us to account for dependence between observations, which often arises when observations are collected from points located in space [29,31]. Ignoring spatial effects in a regression may lead to biased estimates of the model parameters [32]. Examples of such a dependency between the variable of interest and an outcome include pollution levels, health outcomes [30,33], and even economic and socio-demographic variables [31].

Spatial econometric methods have been applied to various health conditions, both communicable and noncommunicable, in a growing body of literature [33]. However, previous studies in the AMR fields have not employed spatial models. To the best of our knowledge, only in one article the authors traced the origin of unknown meat samples and estimate the relative risk of AMR through spatial modeling [34]. AMR infections can be transmitted through direct or indirect contact between humans and animals, which implies that spatial models are relevant for this field of study.

We employed a Spatial Durbin Model (SDM) since it can address our three main objectives:

(1) To explore the effect of AMU in farmed animals on AMR in humans in a country.
(2) To assess how policy actions in one country can influence the AMR situation in neighboring countries.
(3) To examine if AMR can spread over the national borders.

The SDM is a spatial econometric model incorporating the Spatial Autoregressive model (SAR) and the Spatial Lag model (SLX) features. It assumes that the dependent variable is influenced by its spatial lag and the spatial lags of the independent variables. Less intuitively, the SDM can also incorporate the Spatial Error Model (SEM), which accounts for the spatial dependence among the error terms [35]. The standard specification for an SDM is as follows [29,31]:

(1) $$y_{\mathrm{it}}=\rho \omega y_{it}+{\mathrm{\beta x}}_{\mathrm{it}}+\delta \omega x_{\mathrm{it}}+u_{i}+\lambda_{t}+\varepsilon_{\mathrm{it}}$$

Where $y_{\mathrm{it}}$ and $x_{\mathrm{it}}$ are the dependent and independent variable (i means the individuals, and *t* is the time). ρ is a spatial parameter and when it is significant, it is proved that there is a significant spatial dependence among the dependent variables. In contrast, the significant δ, another spatial parameter, demonstrates the existence of a significant spatial dependence among the independent variables. The values of ρ and δ reflect the degree of spatial dependence. Suppose ρ and δ are both zero; the spatial error term may account for the spatial dependence of unobserved factors that affect the observations but are not included in the model. ω is a weighted matrix that defines a spatial neighborhood via a geographic relationship between locations [32,36]. Spatial models integrate space and spatial correlation into mathematics through a spatial weight matrix, an n×n positive matrix with elements $\omega_{\mathrm{ij}}$ at locations i and j. The values of $\omega_{\mathrm{ij}}$, the weights for each pair of observations (based on contiguity or distance rules), define the spatial relations among observations, which is zero for the diagonal elements [37]. $u_{i}$ is an unobserved individual effect, $\lambda_{t}$ is an unobserved time effect, and $\varepsilon_{\mathrm{it}}$ is a random error term. We used *StataSE17*, which uses the *Quasi–Maximum Likelihood (QML)* method for fitting *spatial* panel models.

### 2.2 Data and variables

This section first outlines the scope of the study, which covers our sample and the timeframe. Next, we define the variables and data that we used for the modeling, including the data's sources, measurements, and transformations.

#### 2.2.1 Scope of the study

**Fig. A1:**
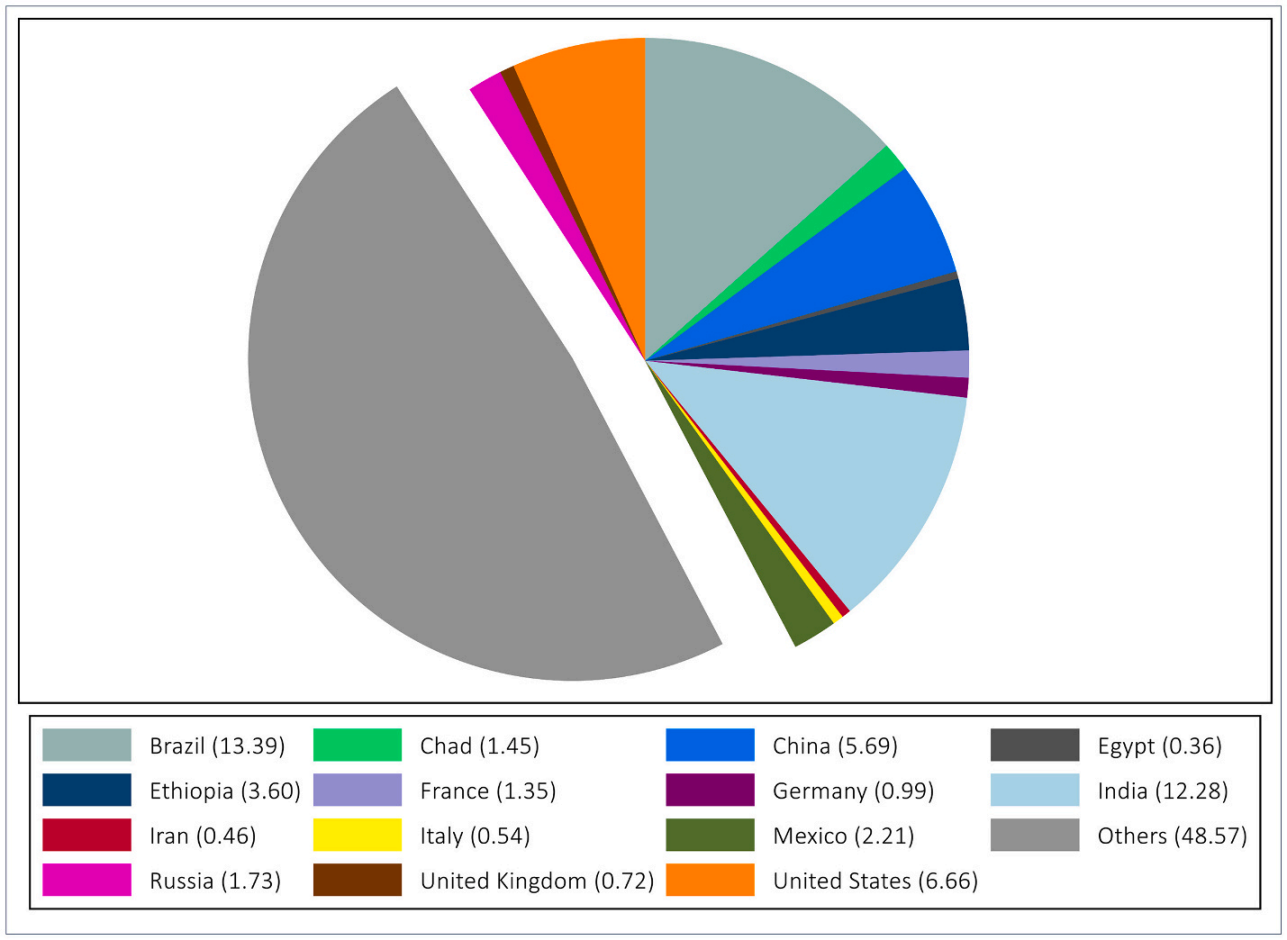
The largest cattle producer countries selected for the study (average from 2010 to 2020), the numbers in parentheses are share (%) of each country in the global production, others excluded; Source: [44] and own calculation

**Fig. A2:**
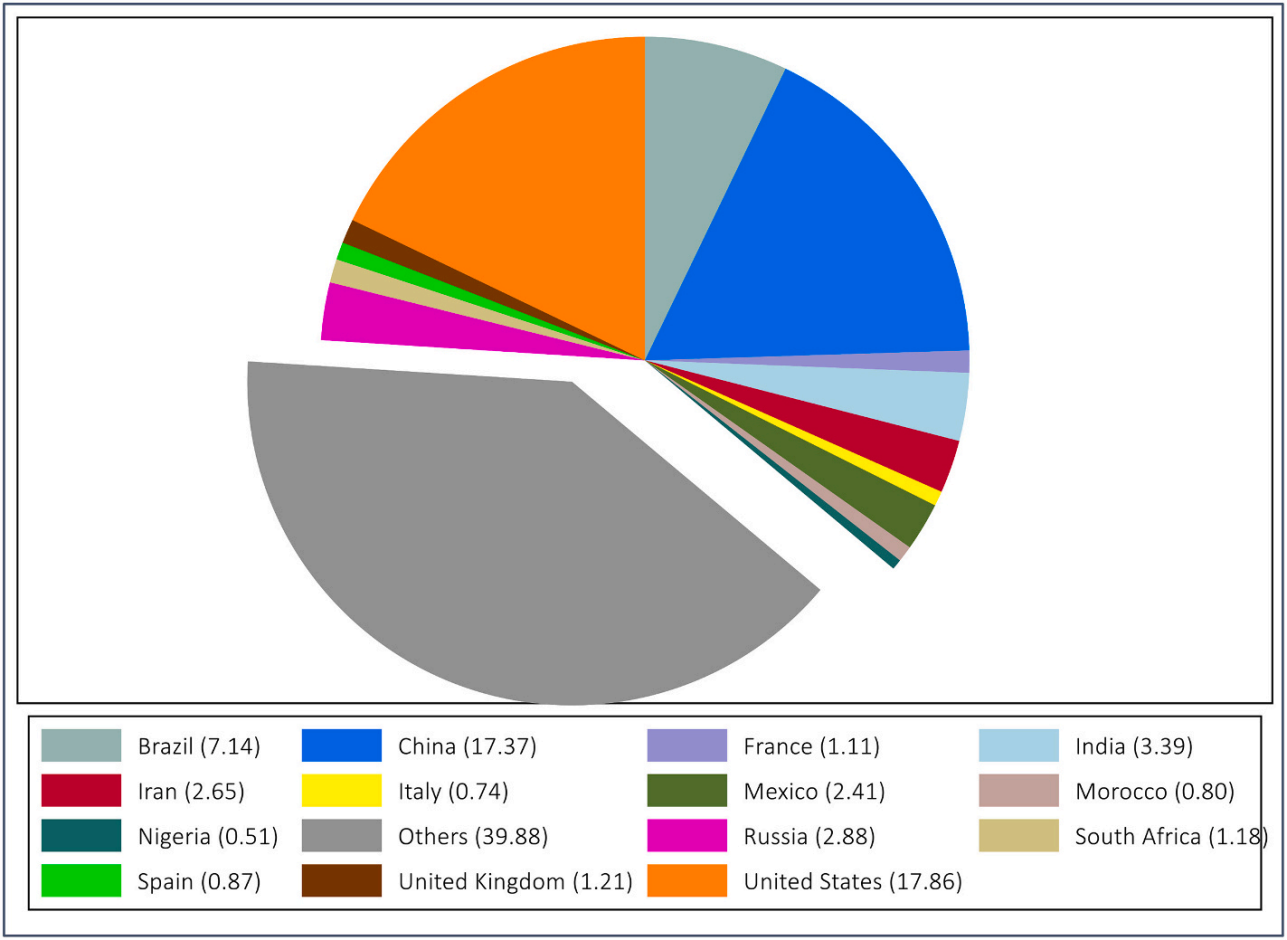
The largest chicken producer countries selected for the study (average from 2010 to 2020), the numbers in parentheses are share (%) of each country in the global production, others excluded; Source: [44] and own calculation.

**Fig. A3:**
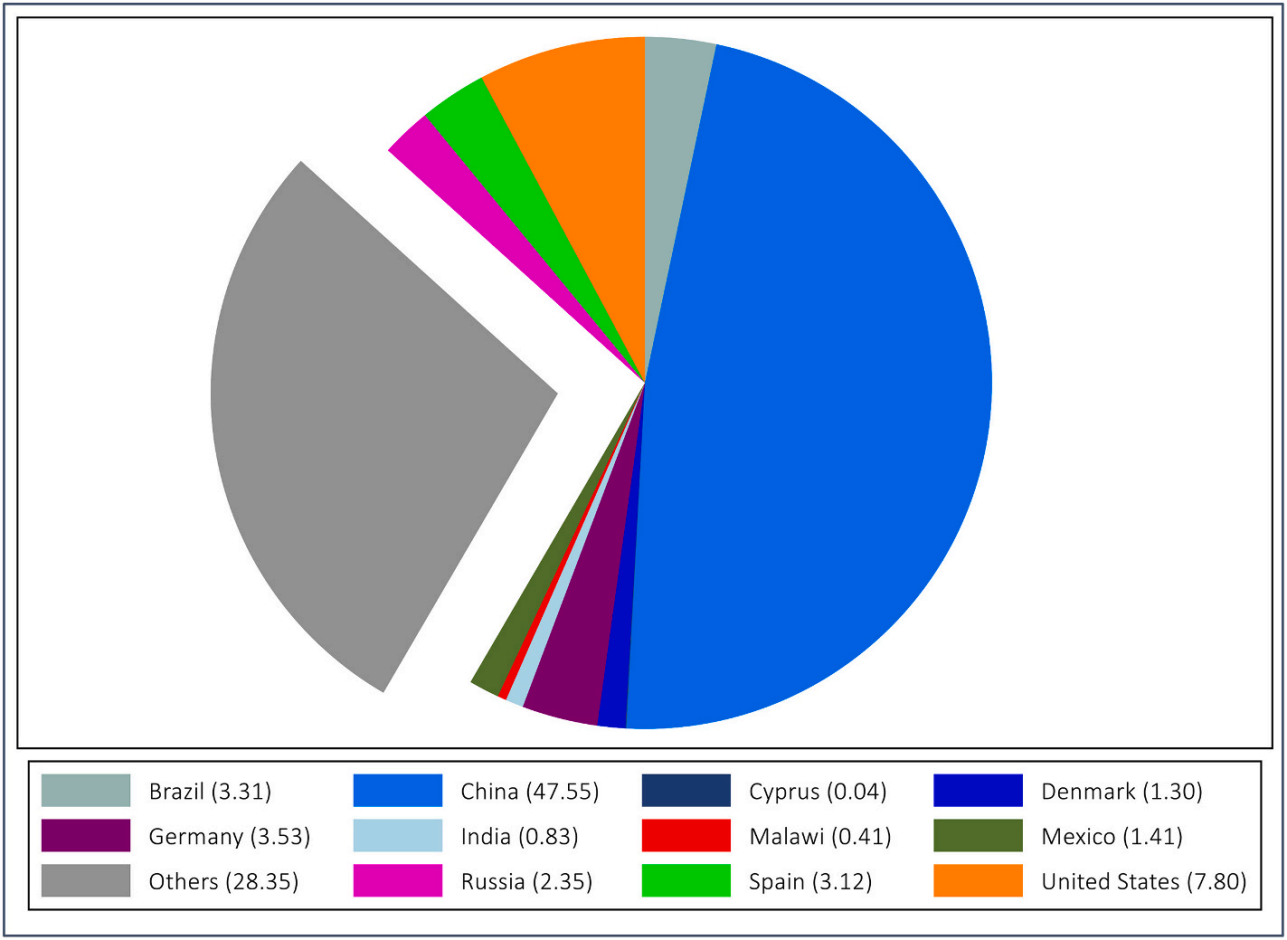
The largest pig producer countries selected for the study (average from 2010 to 2020), the numbers in parentheses are share (%) of each country in the global production, others excluded; Source: [44] and own calculation.

**Fig. A4:**
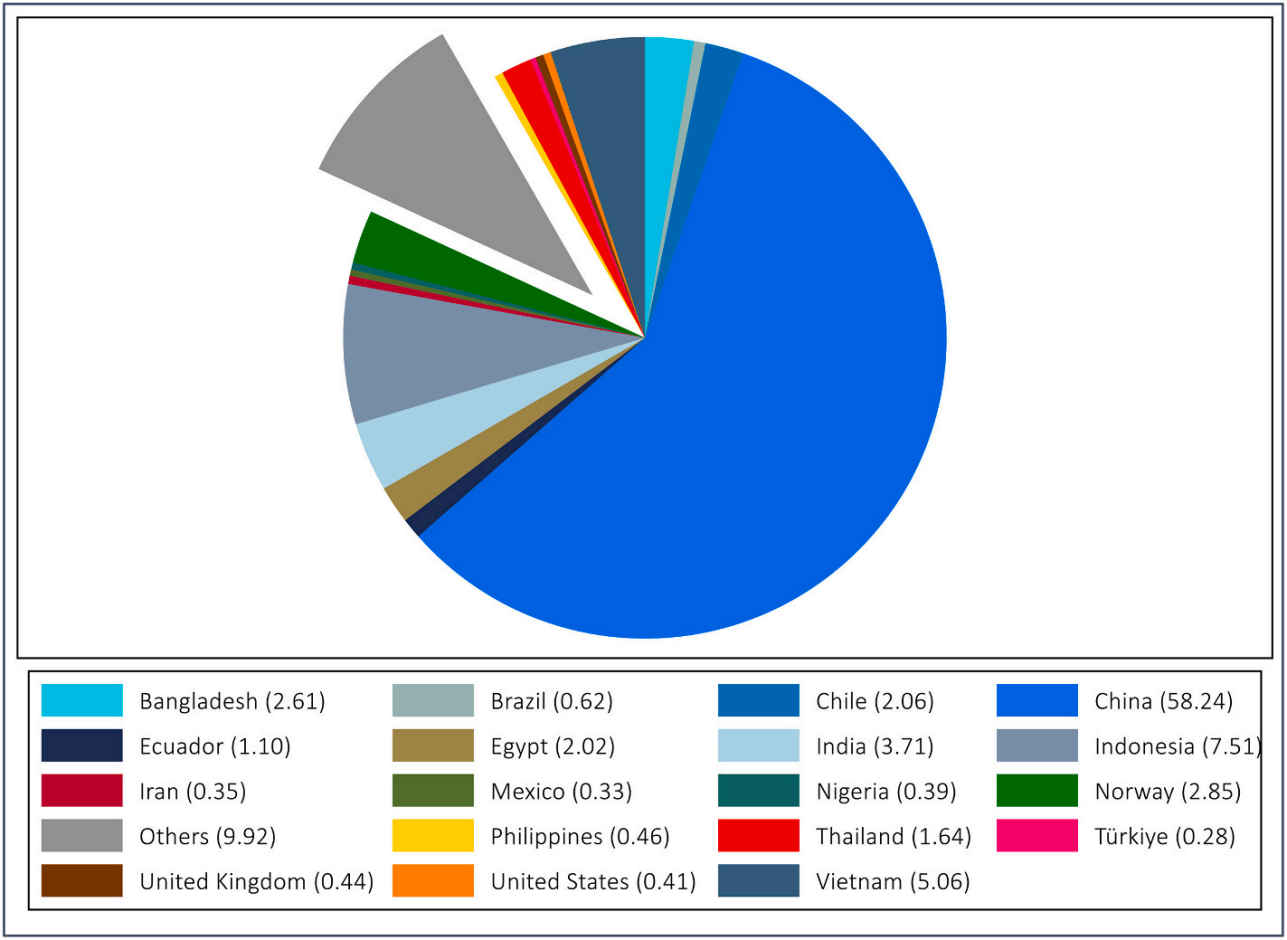
The largest aquatics producer countries selected for the study (average from 2010 to 2020), the numbers in parentheses are share (%) of each country in the global production, others excluded; Source: [45] and own calculation.

We used publicly available data to determine the largest animal producing countries in each region of the world (i.e., World Bank regions including East Asia and the Pacific, Europe and Central Asia, Latin America and the Caribbean, The Middle East and North Africa, North America, South Asia, and Sub-Saharan Africa) for cattle, chickens, pigs, and aquatics (carp, catfish, salmon, shrimp, tilapia, and trout). We collected data on production (2010 to 2020) from the online database of the Food and Agriculture Organization of the United Nations (FAO). Figs. A1 to A4 in Appendix A show the selected producer countries and their related share in global production. The selected countries were thirty and produced 51% of the world's cattle, >60% of chickens, >70% of pigs, and >90% of aquatics. They can also be considered the largest consumers of veterinary antibiotics.

#### 2.2.2 Dependent variable

The Multi-Antibiotic Resistance (MAR) index [38] for *E. coli* and *S. aureus* in humans was calculated as the ratio between the number of resistant bacterial isolates and the total number of isolated tests (Eq. 2) and included in the model as the dependent variable (named AMR in humans).

Data on *E. coli* and *S. aureus*'s antibiotic resistance were collected from the Center for Disease Dynamics, Economics and Policy (CDDEP) online database [39]. Since the European Centre for Disease Prevention and Control (ECDC) considers an AMR level of 20% or more concerning [40], we built the model with resistance levels of 20% or higher.

(2) $$\mathrm{MAR}=\frac{\sum_{1}^{n}R\times N}{\sum_{1}^{n}N}\times 100$$

where:

R is resistant to antibiotic.

N is the number of the isolated tests.

n is the number of antibiotics.

**Fig. 1:**
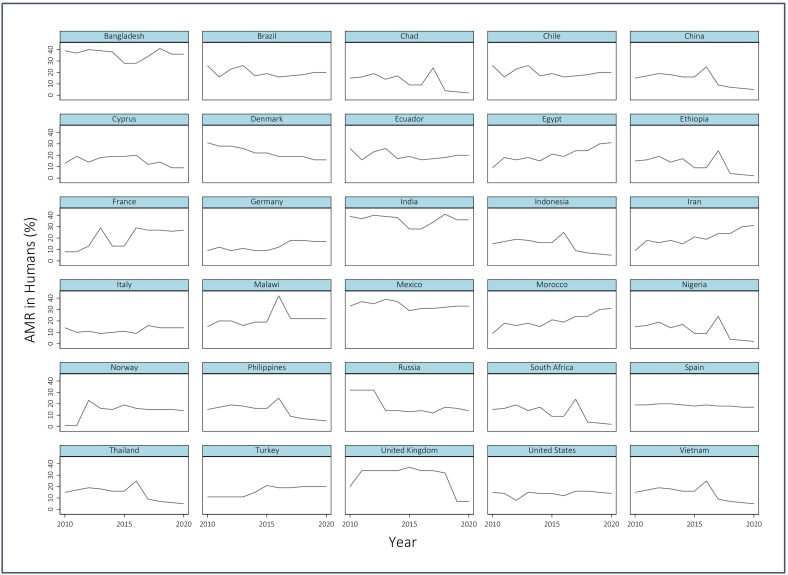
AMR in Humans (%) from *E. coli* and *S. aureus* in the largest animal producing countries (from 2010 to 2020); Source: [39] and own calculation.

Fig. 1 shows the level of the calculated AMR in humans within the largest animal producing countries included in the statistical analysis. AMR in 2019 and 2020 are estimated values (using linear regression and based on data from 2000 to 2018).

#### 2.2.3 Independent variables

While there are numerous contributing factors to AMR, we used the WHO's definition of primary causes of AMR in humans to determine the independent variables for the analysis. According to the WHO, the main drivers of AMR include “*misuse and overuse of antimicrobials; lack of access to clean water, sanitation, and hygiene (WASH) for both humans and animals; poor infection and disease prevention and control in healthcare facilities and farms; poor access to quality, affordable medicines, vaccines, and diagnostics; lack of awareness and knowledge; and lack of enforcement of regulation”* [41].

Based on the above definition, we have defined the following independent variables to use in our empirical model:

(1) We defined two variables for *misuse* and *overuse* of antimicrobials: AMU in humans and AMU in farmed animals. We obtained the data regarding AMU in humans from the online database of CDDEP and estimated (2016–2020) using a linear regression [42]. Regarding AMU in farmed animals, first, we normalized the livestock numbers collected from FAO to a Population Correction Unit (PCU). According to the European Medicines Agency (EMA), 1 PCU equals 1 kg of animal biomass calculated by multiplying the number of farmed animals (alive and slaughtered in each period) by the average weight at treatment (AWT), which is 425 kg for cattle, 1 kg for chickens, and 65 kg for pigs [43]. For aquaculture, we assumed the production in kilograms as PCU. Then, for each country, we multiplied the estimated PCU by the quantity of antibiotics administered to animals in mg of active substance per PCU for each chosen species to approximate the total AMU in farmed animals. In this calculation, we used the global mg per PCU estimated by other studies [10,23,24] for each farmed species in 2010 and 2017. For terrestrials, from 2010 to 2016, we multiplied PCU by the value in 2010, and for 2017 to 2020, we multiplied PCU by the value in 2017. For aquatics, the available mg per PCU is for 2017. So, we multiplied the PCU by the estimated mg per PCU in 2017 for the whole period. For the countries (Denmark, France, the United Kingdom, and the United States) where antibiotic sales information was available, we estimated the country values of mg per PCU by dividing the total antibiotic sales (available in 2017) by the country PCU in 2017 and used it for the whole period to calculate AMU in farmed animals in those countries. FAO online database provided data on production and further PCU calculations [44,45].
(2) A dummy variable was created to represent the poor access to WASH infrastructure, using “effect coding^1^”. According to the WHO, poor access to WASH contributes to >800,000 deaths annually in low- and middle-income countries [46]. Thus, the dummy divides the countries the low- and middle-income countries and the high-income countries, applying the World Bank classification [47].
(3) We considered the percentage of GDP used on health expenditures in a country as a representative of poor infection and disease prevention and control in healthcare facilities and farms, and poor access to quality, affordable medicines, vaccines, and diagnostics. The source for this data is the World Bank [48].
(4) We added another dummy variable to the model to evaluate the effect of management policies at the farm level in a country on AMR in humans in that country and neighboring countries. This dummy serves as an indicator of a lack of regulation. One strategy to minimize the AMU in farmed animals is to restrict or reduce the use of antibiotics as growth promoters [49]. In this study, when a country has laws that regulate AMU, such as prohibiting the use of antibiotics as growth promoters in farmed animals, the dummy considers the country regulated, in opposition to non-regulated for others. We made this dummy using “effect coding” and the “difference in difference coding” systems. A way to estimate the effect of a policy intervention is to use difference in difference coding. This method involves creating a variable that captures the interaction between a binary indicator of the policy status (for example, whether a country has banned the use of antibiotics as a growth promoter in animal farming) and a binary indicator of the time (whether a country is part of the first wave of policy implementation or not) [50]. Based on the regulation in force in the EU in 2006, EU countries are grouped into regulated countries for the whole period, plus the United States, which has been regulated since 2017.

The descriptive summary of the variables is reported in Table 1 below, categorized by the two groups of countries as regulated and non-regulated.

**Table 1:** Statistical summary of the variables in regulated and non-regulated countries included in the analysis; Source: own calculation.

Variables	Unit	Obs.	Mean	S.D.	Min.	Max.
*Non-Regulated Countries*						
AMR⁎ in Humans	%	238	0.31	0.11	0.14	0.71
AMU in Humans	DDD⁎⁎	238	6.74	3.62	1.64	22.02
AMU in Farmed Animals	tons	238	2087.47	4654.99	8.86	21,885.02
Health Expenditure (of GDP)	%	238	5.70	2.78	2.41	16.84

*Regulated Countries*						
AMR in Humans	%	92	0.26	0.08	0.15	0.46
AMU in Humans	DDD	92	9.71	2.72	5.67	14.95
AMU in Farmed Animals	tons	92	626.55	1131.48	5.63	5538.70
Health Expenditure (of GDP)	%	92	9.92	2.02	6.45	16.92

⁎ Total resistance observed (> 0%)

⁎⁎ Defined Daily Dose.

## 3 Results

**Fig. 2:**
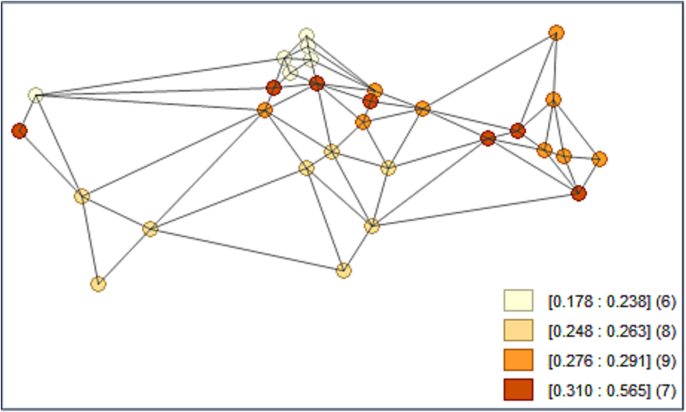
Connectivity map of the weighted matrix over averaged (from 2010 to 2020) AMR in humans in the largest animal producing countries (range numbers in square brackets are level of AMR, and numbers in parentheses are the number of countries located in each category); Source: [39] and own calculations.

The Pesaran-CD test [51] was performed on the dependent variable, AMR in humans, to determine whether neighbors are more correlated than distant ones. As expected, the p-value (CD-test = 3.09; *p*-value = 0.002) close to zero indicates that data are correlated across panel groups. In modeling terms, this behavior displays a significant coefficient on the spatially lagged dependent variable. The outcome allowed us to follow spatial models. Then, a spatial weight matrix was prepared using GeoDa software and used in Stata. The matrix is 30 × 30, row-standardized with zero diagonal factors, and developed via the first-order queen contiguity. Fig. 2 shows the connectivity map of the weighted matrix for the largest animal-producer countries over AMR in humans (on average from 2010 to 2020). As shown in Fig. 2, each country has at least two neighbors, which means the countries are not independent.

Table 2 summarizes the results of spatial panel models (SDM, SAR, and SEM) on AMR in humans. Random effect models specified as fixed effect models were firmly rejected by the Hausman test (Hausman chi^2^ = 3.20; *p*-value = 0.6690). Parameter *ρ* in Table 2 is the coefficient of the spatially lagged dependent variable (AMR in humans), and it is positive and significant in SDM and SAR models. It reveals a significant spatial dependency among AMR in humans between countries, i.e., AMR can transfer over national borders. We expect AMR in humans to correlate with AMU in farmed animals, and AMU in farmed animals in a country is affected by the farming laws in that country. Due to this theory, we added the interaction between AMU in farmed animals in a country and the regulation on AMU in farmed animals in that country (ban on using antibiotics as growth promoters) as a spatially lagged independent variable to the model.^2^ The δ in SDM is the coefficient of this new variable. We expect a negative sign for this variable, meaning that reducing AMU as a growth promoter in a country will reduce AMR in humans in the neighbors. However, this parameter is not significant in our model. The SEM reveals a significant λ parameter. It indicates that the error terms have a spatial dependence, meaning some unobserved factors that are not included in the model as the independent variables influence AMR in humans. However, the magnitude of λ is tiny.

**Table 2:** The Random Effects Spatial Panel Models for AMR in Humans (%); Source: own calculation.

Variables	SDM	SAR	SEM
AMU in Humans (DDD)	0.34^⁎⁎⁎^	0.34^⁎⁎⁎^	−0.11^⁎^
AMU in Farmed Animals (tons)	0.07^⁎⁎^	0.07^⁎⁎^	0.00
WASH (Dummy)	−0.10	−0.11	−0.02
Health Expenditure (% of GDP)	−0.08	−0.10	0.03
Regulation (Dummy)	0.26	0.23	0.13
Intercept	−2.74	−2.66	0.14
δ	−0.01	–	–
ρ	0.34^⁎⁎⁎^	0.34^⁎⁎⁎^	–
λ	–	–	0.05^⁎⁎⁎^
Log-likelihood	−65.21	−65.34	39.43

Significance Levels: ^⁎^ p < 10%, ^⁎⁎^ p < 5%, and ^⁎⁎⁎^ p < 1%.

Partial deviations afford an incomplete understanding of how the independent variables affect the dependent variable in spatial panel models. Henceforth, marginal effects have to be taken into account, which consist of three components: direct, indirect, and total effects. The direct effect measures how a change in an independent variable in one place affects the dependent variable in the same place. The indirect effect measures how a change in an independent variable in one place influences the dependent variable in the neighboring places. The total effect is the sum of the direct and indirect effects. Direct effects are well estimated by the SDM spatial model, even when the model is not correctly specified. However, the model tends to overestimate indirect effects [35]. We proceeded with SDM to assess the direct impact of AMU in farmed animals on AMR in humans, which is our primary aim in this study (Table 3).

**Table 3:** Direct, Indirect, and Total Effects based on SDM; Source: own calculation.

Variables	Direct Effects	Indirect Effects	Total effects
AMU in Humans (DDD)	0.19^⁎⁎^	−0.71^⁎⁎⁎^	−0.52^⁎⁎⁎^
AMU in Farmed Animals (tons)	0.04^⁎^	−0.14^⁎⁎^	−0.10^⁎⁎^
WASH (Dummy)	−0.06	0.22	0.16
Health Expenditure (% of GDP)	−0.05	0.18	0.13
Regulation (Dummy)	0.15	−0.54	−0.40

Significance Levels: ^⁎^ p < 10%, ^⁎⁎^*p* < 5%, and ^⁎⁎⁎^*p* < 1%.

As shown in Table 3, there is a positive correlation between AMU in farmed animals and AMR in humans, suggesting that the *misuse* or *overuse* of antibiotics can contribute to AMR in humans. For a 1% increase of AMU in farmed animals, AMR in humans increases by 0.04%. Similarly, AMU in humans is also positively correlated with AMR in humans, which means that higher levels of AMU in humans lead to higher levels of AMR. For a 1% increase of AMU in humans, AMR in humans increases by 0.19%. Our analysis revealed unexpected evidence about the signs of indirect effects. A solution to this issue is increasing the sample size. Regarding their values, it should also be taken into account that the SDM suffers from an overestimation of indirect effects.

To robustly support our results, we have also evaluated the spatial models on AMR in humans for the 28 EU countries (where data on AMU in farmed animals is available for each country). Due to the unbalanced panel structure of the data,^3^ we fitted the Spatial Lag Model (SLM) and the Spatial Error Model (SEM) (Table 4). The spatial autocorrelation parameters ρ and λ are positive and highly significant with large magnitudes (0.89 and 0.77, respectively). AMU in humans positively affects AMR in humans in the EU based on the two models (SLM and SEM). AMU in farmed animals is only significant in SEM with a tiny coefficient (0.02). Given that the EU countries have strict regulations on AMU in animal farming (prohibition as a growth promoter), AMU's insignificance and tiny coefficient of farmed animals were expected. These findings are consistent with our previous results in Table 2.

**Table 4:** The Spatial Models for AMR in Humans (%) of the EU countries; Source: own calculation.

Variables:	SLM	SEM
AMU in Humans (DDD)	0.47^⁎⁎⁎^	0.45^⁎⁎⁎^
AMU in Farmed Animals (tons)	0.01	0.02^⁎^
Intercept	−4.46^⁎⁎⁎^	−5.70^⁎⁎⁎^
*ρ*	0.89^⁎⁎⁎^	–
λ	–	0.77^⁎⁎⁎^
Log likelihood	−67.22	−73.58
Moran's I	0.076^⁎⁎⁎^	

Significance Levels: ^⁎^ p < 10%, ^⁎⁎^ p < 5%, and ^⁎⁎⁎^ p < 1%.

## 4 Discussion

Even though most antibiotics sold worldwide are used for farmed animals, AMR in humans related to AMU in farmed animals has been understudied and remains unknown. Additionally, although many studies warn about the transmission of AMR between humans, animals, and the environment and that AMR can cross countries' borders, statistical models have not yet confirmed this spreading.

Using spatial analysis, in this study, we explore the connection between AMU in the major farmed animal species and AMR in humans related to two of the most lethal bacteria (*E. coli* and *S. aureus*). The model does not analyze factors that may influence AMR in humans comprehensively. Nonetheless, it focuses on critical variables based on the main drivers of AMR indicated by the WHO.

WHO claims that *misuse* of antibiotics is a primary cause of AMR in both humans and animals. As drawn from CDDEP data [39], the non-response to antibiotics frequently used in animal farming is high in human patients. At the same time, it is low for the antibiotics rarely used on animals. For example, *E. coli* resistance to Aminopenicillins is found at 73.3% (extremely high), while *E. coli* resistance to Glycylcyclines, banned in animal farming, is 0.78% (very low resistance). For *S. aureus*, resistance to Macrolides represents 56.0%, considered very high, while resistance to Vancomycin, a more recent antibiotic banned in animal farming, is very low (0.22%). This evidence shows the importance of avoiding the use of critical antibiotics for both humans and farmed animals.

Another primary cause of AMR in humans, confirmed by the WHO, is the *overuse* of antibiotics in humans and animals. Based on a study in the United Kingdom [52], cattle in non-organic farming use 2.75 times more antibiotics than organic farming. At the same time, pigs and poultry show proportions of 77.5 and 5.7 times higher, respectively. Additionally, data on antibiotics consumption show that Danish pig farms used 38.6 mg per PCU in 2017, 5 times less than the estimated global level (193 mg per PCU). In France, the cattle sector used 11.3 mg of antibiotics per PCU in 2017 versus an estimated global average of 42 mg per PCU, reflecting the effects of regulations [23]. Therefore, reducing non-therapeutic antibiotic treatments in animal farming can be an opportunity to produce with lower AMU. All countries, especially the big producers, should follow such good practices.

Lastly, the estimated model in this study showed a significant spatial dependence (ρ) between countries, which means that resistant bacteria and infectious diseases overcome country borders through traveling and trading, and no country can individually tackle the problem.

One of the main limitations of our study is that we evaluated the level of AMR affecting humans in terms of the percentage of isolated bacterial cultures that tested positive in a given country over one year. The evaluation accuracy depends on the number of tests performed in the country and the extent of the historical series. We extrapolated regional values from the country data, although regions could be differently covered. Some countries involved in the model for their role as relevant producers of commodities of animal origin had not yet have available data regarding tests on isolates. For these countries, we assumed the regional data. Henceforth, these biases might influence the data introduced in the model for the dependent variable (AMR in humans). The model's most relevant independent variable for the analysis is the level of AMU in farmed animals by species. We needed a ten-year historical series from the selected countries for this variable. Such data were available only for France, Denmark, the United Kingdom, and the United States. For the other selected countries, we calculated the total AMU based on FAO data on animal production in the different countries multiplied by the global averages of antibiotic consumption for the various species estimated by other authors [10,23,24]. The lack of data on AMU directly collected in farms might affect the consistency of the model outcomes. However, we validated our results through an estimation of the EU countries.

We recommend future studies to apply the same models to a more countries with different production and consumption levels and roles. Moreover, the models should incorporate a weight matrix that captures the various dimensions of connectivity among countries, such as trade, travel, and other forms of interaction, rather than relying solely on distance.

## 5 Conclusions

This study found a positive association between AMU in farmed animals and AMR in humans using a spatial econometric model. Additionally, the model showed the relevance of AMR spread between countries. Therefore, although the use of antibiotics in farmed animals is currently under control in some countries, many others still need to enforce laws in this area, allowing for unfavorable cross-border effects at the regional and global levels. Health issues such as AMR reveal the interconnectedness of living beings and their shared ecosystems. Thus, more severe actions are necessary at national, regional, and global levels to optimize AMU (*misuse* and *overuse* prevention) in animal farming and harmonize practices in an integrative approach (One Health multisectoral interventions). Examples of husbandry systems that phased out antibiotics suggest that strict limitations on non-therapeutic uses are possible with only minor reductions in productive performance and animal health.

## CRediT authorship contribution statement

**Zahra Ardakani:** Conceptualization, Data curation, Formal analysis, Investigation, Methodology, Software, Validation, Visualization, Writing – original draft, Writing – review & editing. **Massimo Canali:** Funding acquisition, Investigation, Project administration, Supervision, Validation, Writing – review & editing. **Maurizio Aragrande:** Conceptualization, Methodology, Validation, Writing – review & editing. **Laura Tomassone:** Conceptualization, Methodology, Validation, Writing – review & editing. **Margarida Simoes:** Conceptualization, Methodology, Validation, Writing – review & editing. **Agnese Balzani:** Writing – review & editing. **Caetano Luiz Beber:** Conceptualization, Data curation, Formal analysis, Funding acquisition, Investigation, Methodology, Project administration, Supervision, Validation, Writing – review & editing.

## Declaration of Competing Interest

World Animal Protection provided financial support for the research to the Department of Agricultural and Food Sciences of the 10.13039/501100005969University of Bologna. One of the authors is an employee of World Animal Protection. World Animal Protection did not have any role in the study design, collection, analysis, and interpretation of data.

## Data Availability

Data will be made available on request.

## Footnotes

1 Effect coding, unlike dummy coding, which only uses zeros and ones, assigns different weights to the levels of a categorical variable. The sum of the values in any new variable created by effect coding must be zero.

## Footnotes

2 The Xsmle command in Stata lags all the independent variables by default, but we can specify which ones we want to lag using the Durbin option. In our study, we only wanted to lag the interaction variable, because we were interested in its indirect effect. For the other explanatory variables, we focused on their direct effects.

3 Data between 2010 and 2020 are only fully available for some EU countries.
